# Study of Turbulence Promoters in Prolonging Membrane Life

**DOI:** 10.3390/membranes11040268

**Published:** 2021-04-08

**Authors:** Bin Jiang, Binxing Hu, Na Yang, Luhong Zhang, Yongli Sun, Xiaoming Xiao

**Affiliations:** School of Chemical Engineering and Technology, Tianjin University, Tianjin 300072, China; binj@tju.edu.cn (B.J.); diyhoos@foxmail.com (B.H.); yangnayna@tju.edu.cn (N.Y.); zhanglvh@tju.edu.cn (L.Z.); sunyongli@tju.edu.cn (Y.S.)

**Keywords:** turbulence promoter, membrane life, COMSOL simulation, sewage treatment, membrane fouling

## Abstract

Nanofiltration membrane technology is an effective method for secondary treated sewage purification. However, membrane fouling, which is inevitable in the membrane-separation process, can reduce membrane performance and shorten membrane life. Installing a turbulence promoter is a promising means of improving the hydraulic conditions inside the membrane chamber. In this study, the effect of turbulence promoter on prolonging membrane life was studied for the first time. Flat-sheet polyethersulfone nanofiltration membrane was used to filter humic acid solution, used for simulating secondary treated sewage. By comparing photographs and SEM images of the membrane before and after the simulated secondary treated sewage filtration, it was found that humic acid tended to be deposited on the low-velocity region, which was reflected by COMSOL simulation. After incorporating a turbulence promoter, the reduction of the humic acid deposition area and membrane fouling resistance indicated that the turbulence promoter could reduce membrane fouling due to the improved hydraulic conditions. Additionally, the turbulence promoter also increased the flux and reduced the flux decay rate. The turbulence promoter was then place in the crossflow flat-sheet membrane filtration module, and the variation of flux with time was tested in simulated secondary treated sewage with different concentrations. The results showed that the membrane life for the filtration of simulated secondary treated sewage comprising 50, 250, and 500 ppm humic acid increased by 23.1%, 80.4%, and 85.7%, respectively. The results of this article can serve as a reference for the prediction of membrane life and the performance enhancement mechanism of membranes containing a turbulence promoter.

## 1. Introduction

Water shortage and water pollution significantly hinder sustainable development, which is one of the greatest challenges facing humanity in the 21st century. Nanofiltration is a typical pressure-driven membrane-separation process that can treat secondary treated sewage, drinking water, and leachate [1,2,3,4]. Due to the significant improvement of nanofiltration membrane-separation performance and the reduction of operational costs, nanofiltration is widely used to meet the increasing demand of fresh water and strict standards of sewage discharge [5,6]. However, membrane fouling usually results in inefficient membrane processes. The deposition of inorganic compounds, organic macromolecules, and microorganisms on the membrane surface and membrane pores can lead to the decline of permeation flux and membrane life, and the increase in energy consumption and operational costs [7,8,9]. If membrane fouling causes permeate flux to drop to a certain level, the membrane needs to be rinsed or potentially replaced, which not only increases the operational cost, but causes the interruption of the membrane process, thus limiting the application of membrane technology. Researchers are devoted to mitigating the membrane fouling rate as much as possible, thereby prolonging the membrane life [10].

Membrane fouling is a complex phenomenon, and is affected by many factors [11,12,13,14]. These factors can be roughly divided into three categories: solution characteristics, membrane characteristics, and hydraulic conditions. The main challenge facing membrane separation at present is to reduce membrane fouling [15,16,17]. In the long-period operation of the membrane module, contaminants can gradually deposit on the membrane surface, and the deposition layer greatly changes the surface characteristics of the pristine membrane. In this case, the factor affecting membrane fouling is no longer dominated by the pollutant-membrane interaction, but the pollutant-pollution sediment layer interaction [18,19]. The antifouling performance of the pristine membrane will not be effective, and the membrane fouling can only be reduced by improving the hydraulic conditions inside the membrane module. The turbulence promoter (also known as spacer) is an important part of the crossflow membrane filtration module, which can not only provide space for the flow between the membrane envelopes, but also ensures the flow is turbulent [20].

Numerous studies have been undertaken on the preparation of antifouling membranes [21,22,23,24], but reducing membrane fouling from the perspective of membrane modules remains less explored. In recent years, studies have also proposed changing the hydraulic conditions in the membrane chamber using a turbulence promoter to reduce concentration polarization and increase the mass transfer rate [25,26]. Computational fluid dynamics (CFD) is a tool that can be used to intuitively study the fluid flow, particularly the local micro-scale fluid flow phenomenon. Researchers have used CFD to study the fluid flow characteristics of wastewater treatment systems to optimize the design of wastewater treatment units, such as aerated grit tanks [27], multichamber tanks [28,29], oxidation ditches [30], grit chambers [31], bioreactors [32,33], membrane reactors [34,35], and membrane modules [36,37]. Numerous studies [38,39,40,41,42,43,44,45,46] have aimed at understanding the complex flow phenomena occurring in narrow flow channels with a turbulence promoter. Numerous researchers have also conducted two-dimensional [47,48,49] and three-dimensional [50,51,52,53,54] CFD studies of turbulence promoters with different shapes and structures. However, these results lack experimental verification. Srimanta et al. [55,56] used an ultrafiltration membrane to filter juice with a turbulence promoter to explore the correlation between the deposition profiles and the enhancement of flux. The results indicated that turbulence promoters could decrease the deposition thickness and improve the permeate flux. Ferreira et al. [57] evaluated the performance of cylindrical turbulence promoters for the ultrafiltration of purple araça fruit extract with a combination of experiments and simulations, demonstrating that the use of turbulence promoters effectively increased the permeation flux by up to 78%. However, to the best of our knowledge, the effect of turbulence promoters on membrane life has not been studied.

The main purpose of this study is to understand the effect of a turbulence promoter on nanofiltration membrane performance and, in particular, on prolonging the membrane life. First, membrane fouling experiments were carried out to investigate the pollution of simulated secondary treated sewage on the membrane surface, and the detailed flow field characteristics inside the membrane chamber were simulated by CFD. Then, more experiments and simulations without and with a turbulence promoter were conducted to determine the effect of the turbulence promoter on membrane fouling, flux, and membrane resistance. Finally, long-term cyclic filtration experiments with different concentrations of simulated secondary treated sewage (humic acid concentration of 500, 250, and 50 ppm) were carried out to study the effect of the turbulence promoter on prolonging membrane life.

## 2. Experiment

### 2.1. Materials

Commercial polyethersulfone nanofiltration membranes (Molecular weight cut off = 550 Da) were supplied by Zhongli Filtration Equipment Factory (Haining, Zhejiang, China). Humic acid (HA, AR) was obtained from Aladdin Reagent Company (Shanghai, China). Hydrochloric acid (HCl, AR) and sodium hydroxide (NaOH, AR) were purchased from Kermel Chemical Reagents Co., Ltd. (Tianjin, China). Sodium hypochlorite (NaClO, AR) was purchased from FengChuan Chemical Reagent Co., Ltd. (Tianjin, China). Deionized water was used in all experiments.

The turbulence promoter used in the experiment was made by 3D printing, and the printing material was the photosensitive resin 9000E, which has excellent chemical resistance and good thermal stability. The filament diameter of the turbulence promoter was 0.35 mm, and the spacing between two adjacent filaments was 2.8 mm.

### 2.2. Simulated Secondary Treated Sewage

Organic matter is one of the main pollutants in secondary treated sewage nanofiltration treatment [58,59]. Organic matter may also negatively affect the performance of the nanofiltration treatment process [60]. HA is a kind of dissolved natural organic matter in aqueous systems, and has been widely used as a model pollutant by many researchers because of its easy availability and well-characterized properties [61,62,63]. In this study, to study the effect of the turbulence promoter on extending the membrane life through laboratory-scale experiments, HA was used as a model organic pollutant, and different concentrations of simulated secondary treated sewage (SSTS) were prepared by dissolving HA in deionized water to accelerate the aging process of the membrane. The pH value of SSTS was adjusted by adding hydrochloric acid (HCl, 40 wt.%) and sodium hydroxide (NaOH, AR). The flux under SSTS with different pH and HA concentrations was measured by experiments, which provided guidance for the preparation of SSTS in the follow-up experiments (see Figure S1).

### 2.3. Experimental Equipment and Method

**Figure 1:**
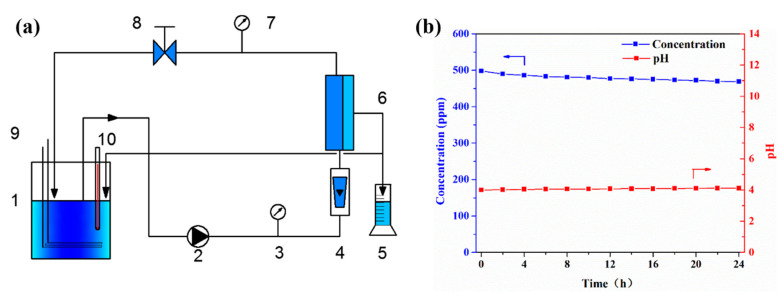
(**a**) Schematic diagram of crossflow membrane filtration equipment ((1) feed tank, (2) plunger pump, (3) pressure gauge, (4) rotameter, (5) volumetric cylinder, (6) membrane chamber, (7) pressure gauge, (8) flow valve, (9) water-cooled heat exchange tube, (10) digital thermometer); (**b**) the variations of concentration and pH of simulated secondary treated sewage (SSTS) in the feed tank during the 24-h filtration experiment.

A schematic diagram of the crossflow flat-sheet membrane filtration equipment used in this study is shown in Figure 1a. The flat-sheet nanofiltration membrane (7.0 × 13.0 cm) was loaded in the membrane chamber. The plunger pump and flow valve were used to obtain the required transmembrane pressure, and the rotameter was used to measure the flow through the membrane chamber. Two pressure gauges were used to measure the pressure drop of the membrane chamber. The temperature of the SSTS was maintained at 25 ± 1 °C by the water-cooled heat exchange tube. The permeated liquid flowed back into the feed tank to keep the concentration and pH of the SSTS approximately constant; this is confirmed in Figure 1b.

Before conducting the membrane experiment, the flat-sheet nanofiltration membrane was soaked in deionized water for 24 h to remove impurities on the membrane surface, and then pre-compacted with deionized water under 0.5 MPa for 1 h to obtain a steady flux. After each experiment, the crossflow filtration equipment was thoroughly cleaned with sodium hypochlorite solution (NaClO, 300 mg/L) to eliminate the interference of the equipment pollution on the experimental results. During the long-term cyclic filtration experiment, the contaminated membrane was rinsed between each cycle with deionized water at a transmembrane pressure (TMP) of 0.3 MPa for 30 min to remove reversible contaminants deposited on the membrane surface.

**Figure 2:**
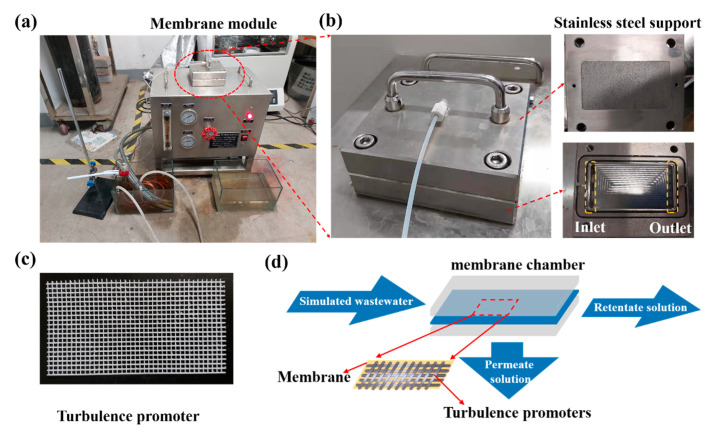
Photographs of (**a**) the crossflow membrane filtration equipment, (**b**) flat-sheet membrane chamber (composed of stainless steel support, inlet, and outlet), and (**c**) turbulence promoter, and (**d**) schematic diagram of the interior components of the membrane chamber.

Figure 2a,b shows the photographs of the crossflow filtration equipment and the interior components of the membrane module. The turbulence promoter was placed in the membrane chamber and the size of the turbulence promoter was 7.0 × 13.0 cm to fit the membrane chamber (Figure 2c,d).

### 2.4. Variable Definitions

The Reynolds number (*Re*) is defined as:

(1) $$Re=\frac{\rho uD}{\mu }$$

where *D* represents hydraulic diameter (m), and ρ, *u*, and μ refer to the density (kg/m^3^), velocity (m/s) and viscosity (Pa·s) of the fluid, respectively. The hydraulic diameter (*D*) can be calculated by Equation (2):

(2) $$D=\frac{4A}{L}$$

where *A* is the area section of the membrane chamber (m^2^), and *L* is the wetted perimeter of the membrane chamber (m).

The shear rate (γ) was calculated using Equation (3):

(3) $$\gamma =\frac{v}{x}$$

where *v* refers to the velocity of the moving layer (m/s), and *x* is the distance between the layers (m).

Flux (J) was calculated by Equation (4):

(4) $$J=\frac{V}{A\cdot \Delta t}$$

where V is the volume of permeate (L), A represents the effective filtration area (m^2^), and Δt represents filtration time (h).

Normalized flux was defined as:

(5) $$Normalized\;flux=\left(\frac{J}{J_{i}}\right)\times 100\%$$

where *J_i_* refers to the initial flux (L/(m^2^·h)).

The flux recovery rate (*FRR*) can measure the antifouling performance of the membrane. The higher the *FRR*, the better the antifouling performance of the membrane. *FRR* was calculated by the following equation:

(6) $$FRR=\frac{J_{R}}{J_{i}}\times 100\%$$

where *J_R_* is the flux (L/(m^2^·h)) measured after membrane rinsing.

Membrane resistance was calculated according to Darcy’s law filtration model, and the formulas are shown in Equations (7)–(11):

(7) $$J=\frac{\Delta P}{\mu R}=\frac{\Delta P}{\mu \left(R_{m}+R_{f}\right)}$$

(8) $$R_{m}=\frac{\Delta P}{\mu_{w}J_{0}}$$

(9) $$R_{f}=\frac{\Delta P}{\mu J}-R_{m}=\frac{\Delta P}{\mu J}-\frac{\Delta P}{\mu_{w}J_{0}}$$

(10) $$R_{irre}=\frac{\Delta P}{\mu J_{R}}-R_{m}$$

(11) $$R_{re}=R_{f}-R_{irre}$$

where *R* is the total resistance, *R_m_* is membrane resistance, *R_f_* is membrane fouling resistance, *R_re_* is reversible membrane fouling resistance, and *R_irre_* is irreversible membrane fouling resistance. Δ*P* represents the TMP (MPa), *J*_0_ is the pure water flux (L/(m^2^·h)) of the membrane, *J* is the flux (L/(m^2^·h)) of the membrane filtering SSTS, and *µ_w_* and *µ* represent the viscosity (Pa·s) of deionized water and SSTS, respectively (viscosity of water and SSTS are shown in Table S1).

The membrane life (*T_life_*) was calculated based on the method of normalized flux decline [64]. However, the practical membrane life is always counted using the unit of years. We used methods similar to those of Brehant et al. to accelerate the aging process of the membrane to make it much easier to study membrane life through laboratory-level experiments [65,66]. In industrial applications, the membrane usually needs to be rinsed or replaced when flux drops by more than 15%. Therefore, in the cyclic filtration experiment, when normalized flux was less than 85%, we paused the experiment to rinse the membrane. We then continued the filtration experiment and repeated the rinse process until the normalized flux of the rinsed membrane was not able to be recovered to 85%. We then stopped the experiment and *T_life_* was expressed as:

(12) $$T_{life}=\sum_{i=1}^{i=n}T_{i}$$

where *T_i_* is the time of the *i*-th filtration cycle, and *n* is the number of the filtration cycle.

### 2.5. Analytical Methods

The surface morphology of the nanofiltration membrane was characterized by Hitachi S-4800 field emission scanning electron microscope (Japan) and Hitachi Regulus8100 field emission scanning electron microscope (Japan). The concentration of HA was determined by a UNICO UV-4802 double beam spectrophotometer (USA) at the wavelength of 254 nm using Lambert–Beer’s Law. The pH values of SSTS were measured by an INESA PHSJ-4A PH meter (China). The viscosity of solutions at 25 °C was measured by a Brookfield DV-II+Pro rotational viscometer (USA).

## 3. COMSOL Simulation

**Figure 3:**
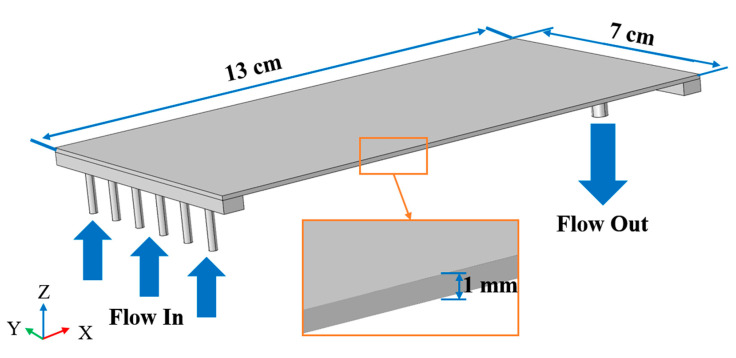
Three-dimensional schematic view of the computational domain.

The fluid flow behavior in the membrane module has an important effect on the membrane performance, and the application of CFD helps to clarify the hydraulic conditions inside the membrane chamber. In this study, 3D CFD simulations were performed to reveal the fluid flow behavior in the membrane chamber. A commercial CFD software package COMSOL Multiphysics 5.4 (COMSOL Inc., Stockholm, Sweden) was used, which employs the finite element method. To ensure the accuracy of the simulation, the inlet flow rate and physical parameters in the simulation were consistent with the actual experiment. Figure 3 shows the membrane chamber model used to perform simulations, and the specific model parameters are shown in Table 1.

**Figure 4:**
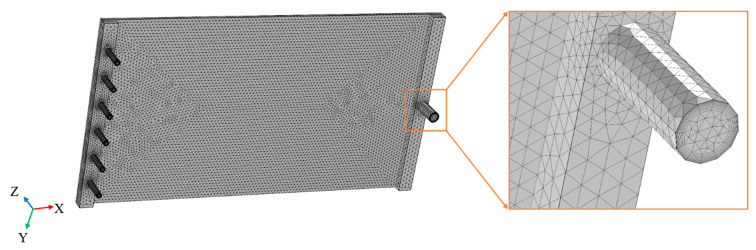
Mesh situation of the membrane chamber.

The applied mesh was a free tetrahedral mesh generated by considering the mechanism controlled by physics. To better capture the fluid flow at the boundary layer, the eight-layer boundary layer mesh was divided (Figure 4). After studying the mesh independent (see Figure S2), a mesh resolution of 609,555 cells (fine grid) was chosen for the research. This work did not study the microfluidic flow inside the membrane, so the membrane was considered to be a two-dimensional smooth plane. Given that the membrane permeate velocity was much lower than the crossflow velocity, no-slip wall conditions were applied on the membrane surface and membrane chamber walls [67]. In this simulation, the fluid flowed through the inlet tube, membrane chamber, and outlet tube successively. Without the turbulence promoter, the Reynolds numbers of different parts were different (*Re* = 17,977, 4765, and 53,932) due to the different flow velocities and hydraulic diameters of different parts. When the turbulence promoter was used, the turbulence promoter occupied the flow space in the membrane chamber, leading to an increase in the flow velocity and an increase in the Reynolds number of the membrane chamber (*Re* increased from 4765 to 7330). It was obvious that the fluids were in a turbulent state within the velocity range selected in this study. The Re-normalization group (RNG) k-ε turbulence model adapts the turbulent transport equation by introducing a damping function, taking into account the influence of eddy currents on turbulence, and is suitable for turbulence simulation when the near-wall effect is significant [68,69]. Therefore, the RNG k-ε turbulence model was selected for calculation. The convergence criterion was 0.0001.

**Figure 5:**
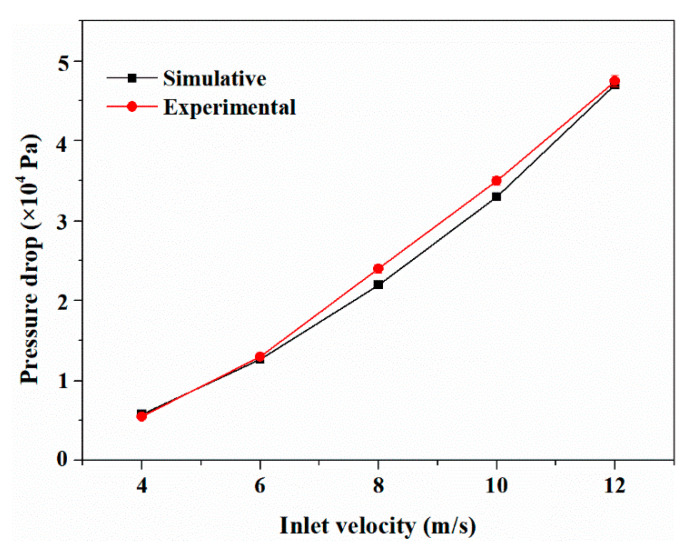
Comparison of simulated and experimental values of axial pressure drop in the membrane chamber.

To validate the accuracy of the CFD calculation model and ensure the reliability of the simulation results, the CFD model was used to calculate the axial pressure drop of the membrane chamber at different inlet flow rates, and the calculated values were compared with the experimental values. The comparison between the simulated and experimental values is shown in Figure 5. It can be seen that the value calculated by the CFD model is consistent with the value measured by the experiment, which verifies the reliability of the model.

## 4. Results and Discussion

### 4.1. Effect of Turbulence Promoter on the Alleviation of Membrane Fouling

**Figure 6:**
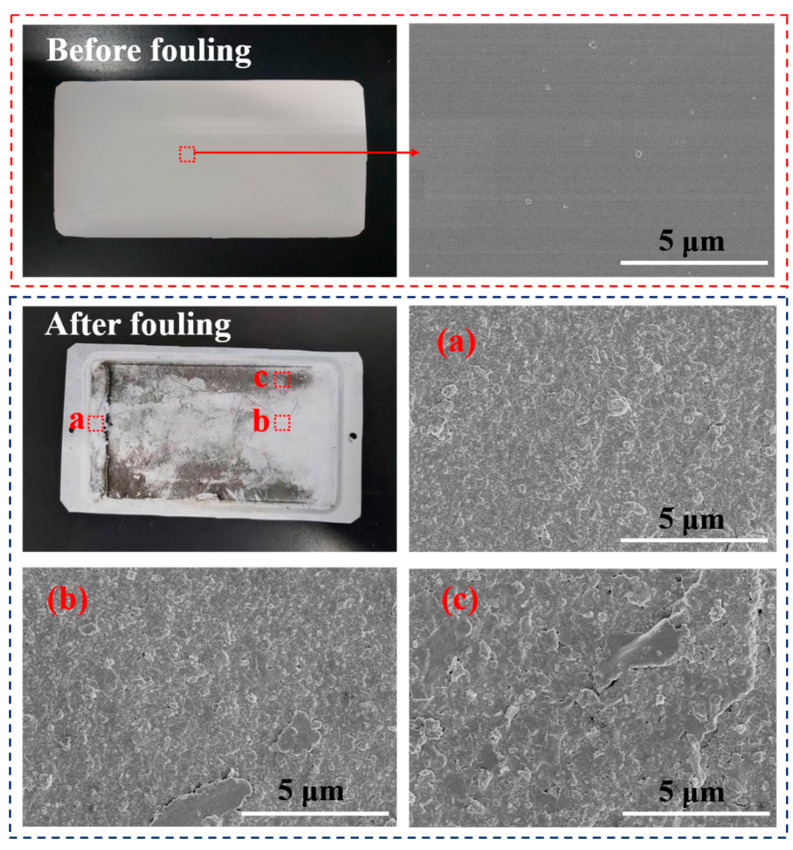
Photographs and SEM images of the membrane before and after fouling by simulated secondary treated sewage (SSTS: 200 ppm HA, pH = 7; TMP = 0.5 MPa).

**Figure 7:**
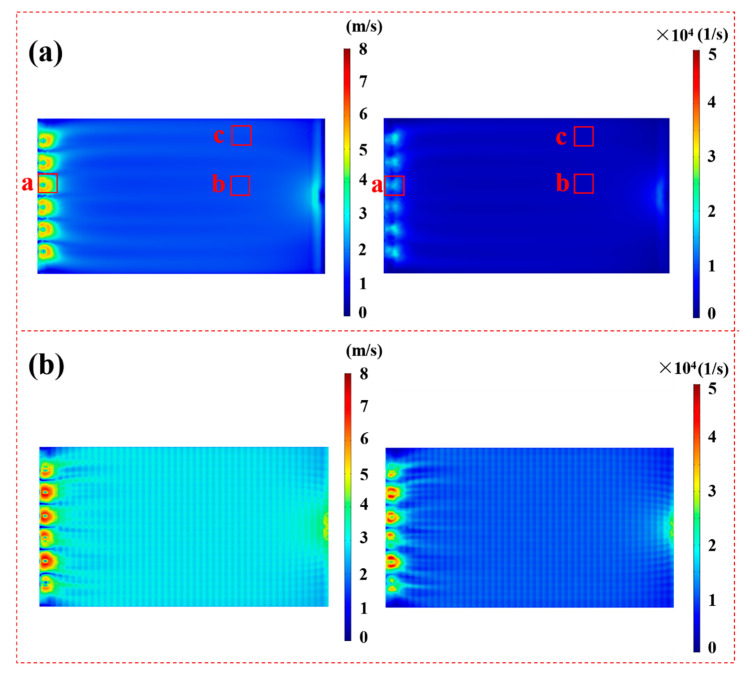
Velocity and shear rate profiles in the membrane (**a**) without the turbulence promoter and (**b**) with the turbulence promoter (inlet velocity = 8 m/s).

By comparing the photographs and the SEM images of the membrane before and after 2 h of fouling by SSTS (200 ppm HA, pH = 7) (Figure 6), it can be seen that HA deposited unevenly on the membrane surface. The level of membrane fouling followed the order Zone c > Zone b > Zone a, where extensive fouling was deposited in Zone c, considerable fouling occurred in Zone b, and no visible fouling was found in Zone a. From the COMSOL simulation results in Figure 7a, it can be seen that the magnitude of the fluid velocity and shear rate on the membrane surface both followed the order Zone a > Zone b > Zone c, which indicates the HA is harder to deposit on the membrane area with higher velocity and shear rate. This may be because a fluid with larger velocity and shear rate is more able to flush the accumulated contaminants onto the membrane surface to flow back into the flow channel. The results showed that the membrane module with improved flow characteristics is an effective strategy to alleviate membrane fouling.

**Figure 8:**
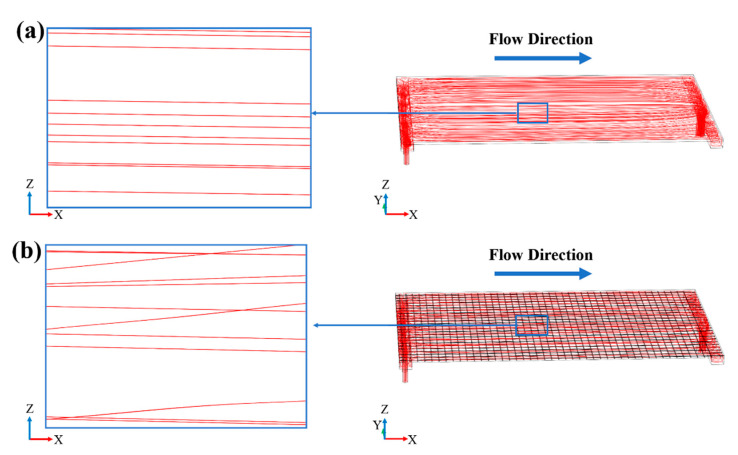
The fluid path line inside the membrane chamber (**a**) without the turbulence promoter and (**b**) with the turbulence promoter (inlet velocity = 8 m/s).

**Figure 9:**
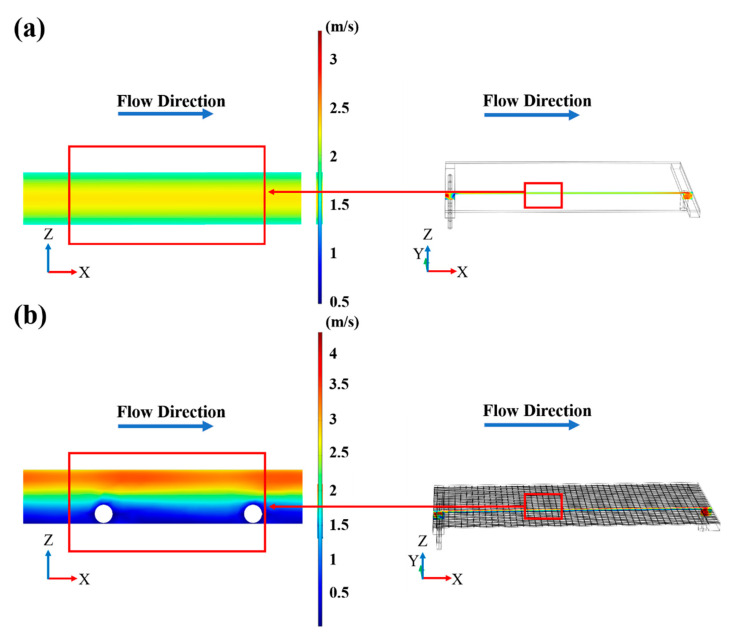
The velocity distribution of the inner section of the membrane chamber (**a**) without the turbulence promoter and (**b**) with the turbulence promoter (inlet velocity = 8 m/s).

According to COMSOL calculation results, after installing the turbulence promoter, the average velocity and average shear rate on the membrane surface increased from 1.77 m/s and 4056 1/s to 2.98 m/s and 11,523 1/s, respectively. Figure 7 also shows that the turbulence promoter leads to a more uniform velocity distribution on the membrane surface. Figure 8 shows the path line of the fluid inside the membrane chamber. It can be seen that the fluid path lines without the turbulence promoter were almost parallel, whereas the fluid path lines with the turbulence promoter obviously intersected, which indicates that turbulence promoter can improve the turbulence and mixing degree of the fluid. Figure 9 shows the velocity distribution of the longitudinal cross-section inside the membrane chamber. It can be seen that the turbulence promoter reduced the area of the flow channel, thereby increasing the flow velocity and reducing the thickness of the laminar boundary layer on the membrane surface. In summary, the circulating flow can improve the flow characteristics in the membrane chamber to prevent particles depositing on the membrane surface.

**Figure 10:**
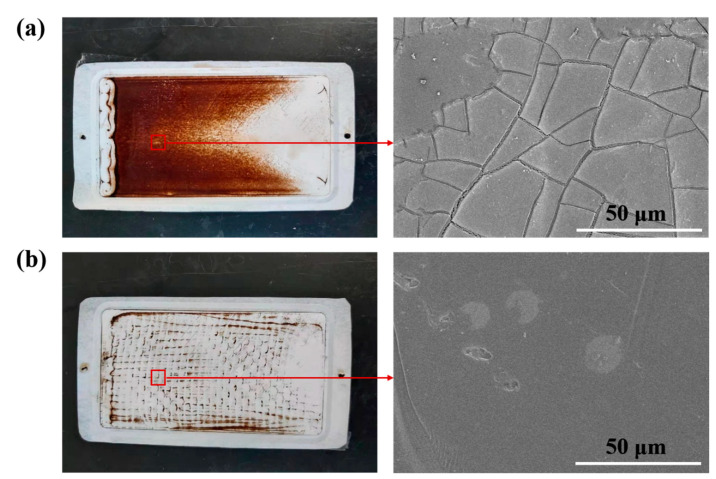
Photographs and SEM images of the contaminated membrane (**a**) without the turbulence promoter and (**b**) with the turbulence promoter (SSTS: 500 ppm HA, pH = 4; TMP = 0.5 MPa).

As indicated in Figure 10a, after 2 h fouling by SSTS (500 ppm HA, pH= 4), very heavy contamination was found on the membrane without the turbulence promoter and about two thirds of the membrane surface was covered by HA deposition. However, Figure 10b shows that the contamination area on the membrane with the turbulence promoter was significantly reduced, and only a small amount of HA was deposited in the dead zone formed by the contact of the turbulence promoter filaments with the membrane surface. The experimental results proved that the turbulence promoter can effectively reduce the deposition of contaminants on the membrane surface.

### 4.2. Effect of Turbulence Promoter on Flux and Membrane Resistance

#### 4.2.1. 100-min Membrane Filtration Experiment

**Figure 11:**
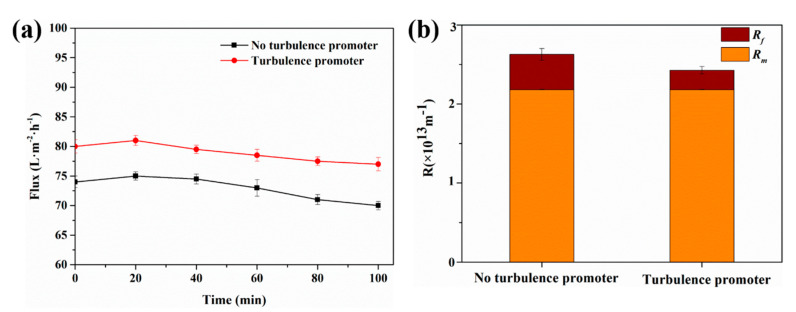
(**a**) Variations of flux with time and (**b**) effect of the turbulence promoter on membrane fouling resistance (SSTS: 500 ppm HA, pH = 4; TMP = 0.5 MPa).

To study the effect of the turbulence promoter on flux, SSTS (500 ppm HA, pH = 4) was used as the feed liquid in the experiment. Filtration experiments were carried out without the turbulence promoter and with the turbulence promoter respectively, and the variations of flux over time were recorded. The results are shown in Figure 11a. It can be seen that the flux increased first and then decreased in both cases, which may be due to the expansion of the membrane pores during the initial filtration stage, thereby increasing the flux, and, as the filtration time increased, pollutants were gradually deposited on the membrane surface and inside membrane pores, thereby reducing flux. The average value of flux without the turbulence promoter was 72.92 L/(m^2^·h), whereas in the case with the turbulence promoter, the average was 78.92 L/(m^2^·h), which was 8.2 ± 1.1% higher than the former. This confirmed that the turbulence promoter can improve the flux [56,57]. Figure 11b shows the effect of the turbulence promoter on membrane resistance. It can be seen that the values of membrane fouling resistance *R_f_* were about 4.48 × 10^12^ m^−1^ and 2.47 × 10^12^ m^−1^, respectively, in the case of without and with the turbulence promoter. The turbulence promoter reduced *R_f_* by 45%, thereby reducing the total resistance *R* by 7.6% from 2.63 × 10^13^ m^−1^ to 2.43 × 10^13^ m^−1^. The experimental results showed that the turbulence promoter can effectively inhibit membrane fouling, reduce membrane fouling resistance, and thus increase flux.

#### 4.2.2. 24-h Membrane Filtration Experiment

**Figure 12:**
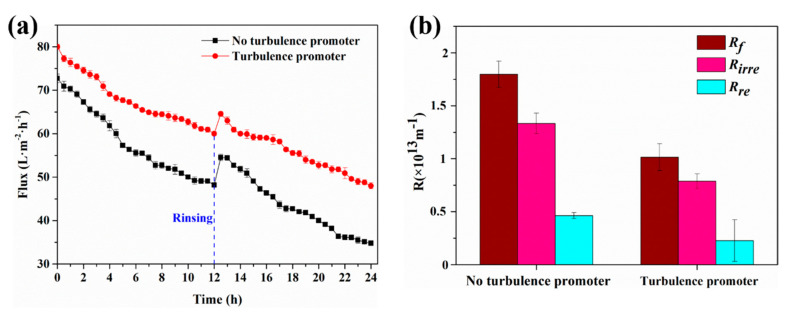
(**a**) Variations of flux with time and (**b**) effect of the turbulence promoter on membrane fouling resistance, irreversible membrane fouling resistance and reversible membrane fouling resistance (SSTS: 500 ppm HA, pH = 4; TMP = 0.5 MPa).

Next, a 24-h membrane filtration experiment consisting of two 12-h cyclic filtration experiments was conducted, and the contaminated membrane was rinsed between the two cycles. As can be seen from Figure 12a, compared with the filtration experiment of 100 min, the flux decreased more obviously in the filtration experiment of 24 h. In addition, in the case with the turbulence promoter, the rate of flux reduction was smaller, which indicates that the turbulence promoter can effectively reduce the flux decay rate. To be specific, for the conventional filtration without the turbulence promoter, the flux decay rates were 33.7 ± 1.2% and 36.1 ± 0.1% in the two cycles, whereas for the case with the turbulence promoter, the flux decay rates were 25 ± 0.8% and 25.6 ± 1.2%. This is mainly ascribed to the fact that the turbulence promoter can reduce the accumulation of foulant particles on the membrane surface. In addition, this can also be explained by the critical flux, which is the maximum stable flux of a given feed solution [70]. Once the actual flux is greater than the critical flux, membrane fouling begins to occur and flux drops. According to the conclusion of Neal et al. [71], the presence of the turbulence promoter leads to a marked increase in the critical flux. Therefore, the turbulence promoter can enhance flux and reduce the flux decay rate.

After a rinsing operation between the two cycles, the calculated flux recovery rate (*FRR*) was 74.9 ± 0.1% and 80.6 ± 0.9% for the membrane without and with the turbulence promoter, respectively, which indicates that the turbulence promoter can improve the antifouling ability of the membrane. As can be seen from Figure 12b, without the turbulence promoter, *R_f_*, *R_irre_*, and *R_re_* were 1.79 × 10^13^ m^−1^, 1.33 × 10^13^ m^−1^, and 0.46 × 10^13^ m^−1^, respectively, whereas with the turbulence promoter, *R_f_*, *R_irre_*, and *R_re_* were 1.01 × 10^13^ m^−1^, 0.79 × 10^13^ m^−1^, and 0.22 × 10^13^ m^−1^, respectively. It is obvious from the data that the turbulence promoter can effectively reduce the membrane fouling resistance.

### 4.3. Effect of Turbulence Promoter on Membrane Life

To study the effect of the turbulence promoter on prolonging membrane life, long-term cyclic filtration experiments with different concentrations of SSTS (HA concentration at 500, 250, and 50 ppm) were carried out.

#### 4.3.1. Experiment with HA Concentration at 500 ppm

**Figure 13:**
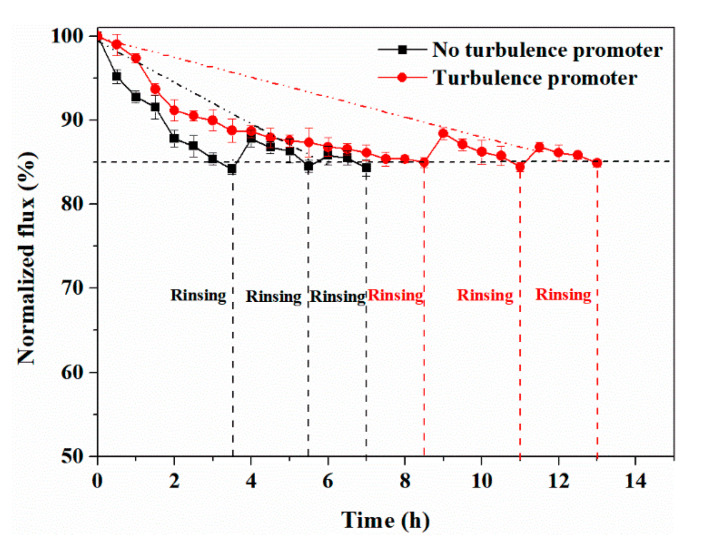
Variations of normalized flux with time (SSTS: 500 ppm HA, pH = 4; TMP = 0.5 MPa).

As observed in Figure 13, the normalized flux for the membrane without the turbulence promoter decreased to 85% after 3 h; the first cyclic experiment was then suspended for membrane rinsing, before the experiment was continued for the next cycle. After the first and second rinsing, the normalized flux recovered to 87.8 ± 0.7% and 85.8 ± 0.8%, respectively. After three cycles, the normalized flux was not able to reach 85%, indicating that the membrane life during experiment without the turbulence promoter was about 7 h. By contrast, the normalized flux for the membrane with the turbulence promoter decreased significantly more slowly. The first cycle was 8.5 h and the normalized flux after three cycles could not recover to 85%, indicating that the membrane life equipped with the turbulence promoter was 13 h, which was 85.7% longer than that of the membrane without the turbulence promoter. Due to the use of the turbulence promoter, the number of cycles increased and the time consumption for each cycle increased because of the decrease in the flux decay rate. Thus, the turbulence promoter can effectively prolong the membrane life and reduce the number of membrane replacements, which is conducive to reducing the operational cost. In addition, it was found that as the number of membrane rinses increased, the *FRR* gradually decreases, which indicates that the gradual increase in irreversible fouling of the membrane leads to a decrease in membrane rinsing efficiency. The *FRR* point was linearly fitted, and the fitting equations of flux recovery rate and membrane life are shown as follows:

(13) $$y=-2.4648x+99.411\left(R^{2}=0.9589\right)$$

(14) $$y=-1.1857x+99.832\left(R^{2}=0.999\right)$$

where *y* and *x* are the flux recovery rate (%) and time (h), respectively. Equations (13) and (14) are linear fitting equations without and with the turbulence promoter, respectively.

According to the linear fitting equation, the calculated membrane lives without the turbulence promoter and with the turbulence promoter were 5.8 and 12.5 h, respectively, which were 17.1% and 3.8% different from the measured membrane lives, respectively. The difference between the calculated value and the measured value is within an acceptable range, indicating that this quick calculation method can be used to predict membrane life in similar experimental systems.

#### 4.3.2. Experiment with HA Concentration at 250 ppm

**Figure 14:**
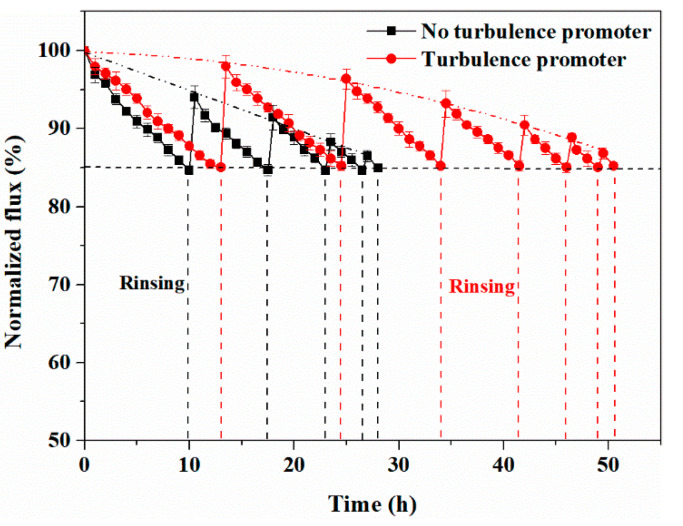
Variations of normalized flux with time (SSTS: 250 ppm HA, pH = 4; TMP = 0.5 MPa).

Figure 14 shows the variation of the normalized flux with time in the SSTS (250 ppm HA, pH = 4). As illustrated in Figure 13, for the case without the turbulence promoter, the cyclic filtration experiment contained five cycles and the *FRR* could not reach 85% after 28 h of the experiment, indicating that the membrane life was 28 h. For the case with the turbulence promoter, the cyclic filtration experiment lasted for seven cycles and the *FRR* could not reach 85% after 50.5 h, indicating that the membrane life was 50.5 h. Hence, for the experiment with HA concentration at 250 ppm, the turbulence promoter increased the membrane life by 80.4%. The fitting equations of the *FRR* with time under the condition of not adding the turbulence promoter and adding the turbulence promoter are Equations (15) and (16), respectively.

(15) $$y=-0.4889x+99.777\left(R^{2}=0.9943\right)$$

(16) $$y=-0.0043x^{2}-0.0443x+99.819\left(R^{2}=0.9958\right)$$

According to the fitting equation, the membrane lives without the turbulence promoter and with the turbulence promoter are 30.2 and 53.8 h, respectively, which differ from the measured values by 7.8% and 6.5%, respectively.

#### 4.3.3. Experiment with HA Concentration at 50 ppm

**Figure 15:**
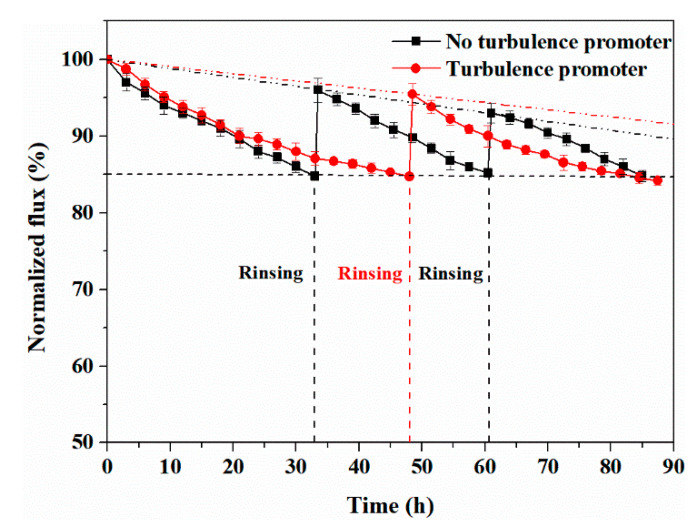
Variations of normalized flux with time (SSTS: 50 ppm HA, pH = 4; TMP = 0.5 MPa).

Figure 15 shows the variation of the normalized flux with time in the SSTS (50 ppm HA, pH = 4). Because the expected experiment time was too long, two 90-h maximum membrane filtration experiments were carried out for this case, and the total membrane life was predicted by the fitting equation. It was found that the flux decay rate for filtering a 50 ppm HA involved SSTS was significantly smaller than that for filtering a higher concentration HA involved SSTS. For the case of the membrane without the turbulence promoter, the time consumption for the first three cycles was 85 h. After two membrane rinses, the *FRR* values were 96 ± 1.1% and 93 ± 0.9%, respectively. The fitting equation for the *FRR* over time is shown in Equation (17), and the predicted membrane life according to this equation is 130 h. For the case of the membrane with the turbulence promoter, the time consumption for the first two cycles was 87.5 h and the *FRR* was 95.5 ± 1.0% after membrane rinsing. The fitting equation of the *FRR* over time is shown in Equation (18), and the predicted membrane life according to this equation is 160 h, which indicates that turbulence promoter prolongs the membrane life by 23.1%.

(17) $$y=-0.1149x+99.953\left(R^{2}=0.9993\right)$$

(18) $$y=-0.0937x+100\left(R^{2}=1\right)$$

## 5. Conclusions

The focus of this research was to explore the effect of turbulence promoters on nanofiltration membrane performance, particularly the membrane life, using a combination of experiments and numerical simulations. It was found that the installation of a turbulence promoter increased the average velocity and shear rate on the membrane by 68.4% and 184.1%, respectively, which resulted in the reduction of HA deposition on the membrane surface. In addition, the turbulence promoter also reduced membrane fouling resistance, increased flux, and reduced the flux decay rate. Consequently, the long-term cyclic filtration experiment results revealed the use of a turbulence promoter could extend the membrane life by increasing the number of cycles and the time consumption for each cycle because of the decrease in the flux decay rate. For filtration of simulated secondary treated sewage comprising 50, 250, and 500 ppm HA, the turbulence promoter increased the membrane life by 23.1%, 80.4%, and 85.7%, respectively. Finally, a quick calculation method containing fitting equations of the flux recovery rate was provided to evaluate membrane life.

## Figures and Tables

**Table 1:** Numerical model parameters.

Parameter	Value	Unit
**Model geometry**		
Chamber length	130	mm
Chamber width	70	mm
Chamber height	1	mm
Inlet pipe diameter	2	mm
Inlet pipe length	10	mm
Outlet pipe diameter	4	mm
Outlet pipe length	10	mm
**Model parameters**		
Inlet flow velocity	8	m/s
Outlet pressure	0	Pa
Water density	997	kg/m^3^
Water viscosity	0.89 × 10^−3^	Pa·s
Temperature	25	°C

## Supplementary Materials

The following are available online at https://www.mdpi.com/article/10.3390/membranes11040268/s1, Figure S1: (a) Flux in simulated secondary treated sewage comprising different concentrations of HA (SSTS: pH = 7), (b) effect of pH on flux (SSTS: 200 ppm HA; TMP = 0.5 MPa). Figure S2: (a) Velocity and (b) pressure at the center line of the membrane chamber under different mesh sizes. Table S1: Viscosity of water and simulated secondary treated sewage at 25 °C.
