# Supplementary material for: Study of Turbulence Promoters in Prolonging Membrane Life
Source: Membranes (Basel). 2021 Apr 8;11(4):268. doi: 10.3390/membranes11040268 (PMC8068148; doi:10.3390/membranes11040268)
Supplement: Supplementary file 1 [file membranes-11-00268-s001.pdf]

Supplementary Material

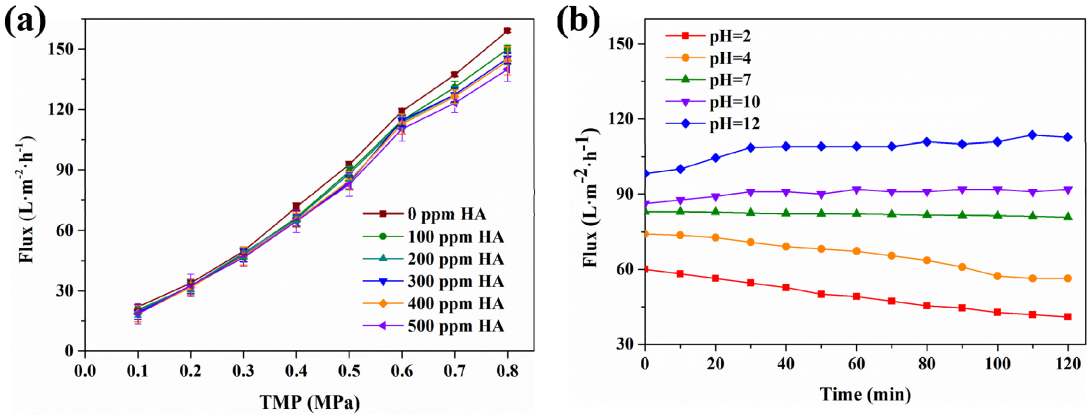

Figure S1. (a) Flux in simulated secondary treated sewage comprising different concentrations of HA (SSTS: pH = 7), (b) effect of pH on flux (SSTS: 200 ppm HA; TMP = 0.5 MPa).

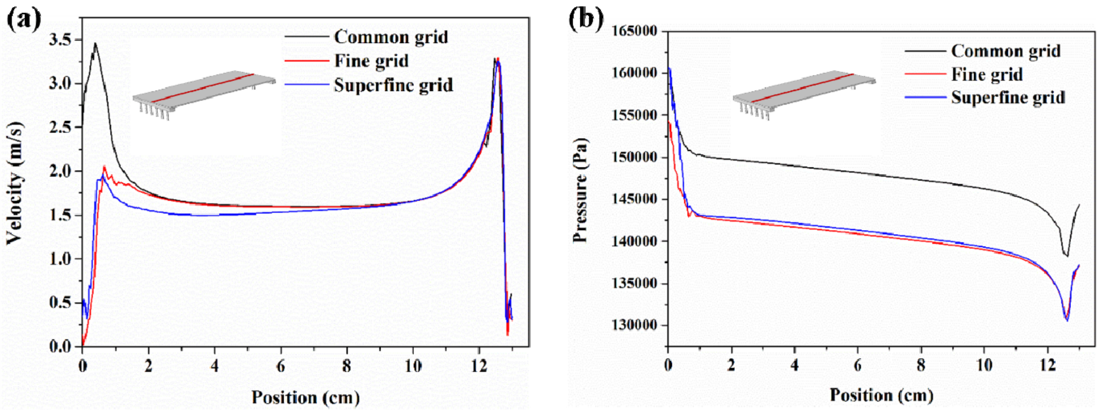

Figure S2. (a) Velocity and (b) pressure at the center line of the membrane chamber under different mesh sizes (inlet velocity = 8 m/s).

Table S1. Viscosity of water and simulated secondary treated sewage at 25°C.

	Viscosity (mPa·s)
water	0.895 ± 0.005
SSTS: 50 ppm HA	0.910 ± 0.0015
SSTS: 100 ppm HA	0.918 ± 0.001
SSTS: 200 ppm HA	0.923 ± 0.002
SSTS: 300 ppm HA	0.929 ± 0.0005
SSTS: 400 ppm HA	0.932 ± 0.001
SSTS: 500 ppm HA	0.940 ± 0.0025
